# Ramifications of Setup Margin Use During Frameless Stereotactic Radiosurgery/Therapy With Gamma Knife Icon Cone-Beam Computed Tomography (CBCT): A Dosimetric Study

**DOI:** 10.7759/cureus.21996

**Published:** 2022-02-07

**Authors:** William N Duggar, Bart Morris, Rui He, Claus Chunli Yang

**Affiliations:** 1 Radiation Oncology, University of Mississippi Medical Center, Jackson, USA

**Keywords:** rational margin, ptv, frameless radiosurgery, setup margin, gamma knife icon

## Abstract

Objective

The objective is to explore the possibility of optimal/rational application of setup margin during treatment planning for frameless stereotactic Gamma Knife radiosurgery/therapy.

Methods

Uncertainty measurements for frameless Gamma Knife Icon treatment were used to calculate the necessary setup margin via four different published recipes and these margins were subsequently applied to treatment plans of 30 previously treated patients and replans were generated meeting comparable plan quality metrics. All plans were then analyzed based on the ability to maintain normal tissue dose tolerances and the relative increase in target dose coverage probability using a pass/fail scoring system based on published normal tissue dose constraints and an in-house developed optimal scoring method.

Results

Gross tumor volume/planning target volume (GTV/PTV) size strongly correlated with both meeting normal tissue tolerances and optimal scores for single fraction plans corroborating published clinical outcomes. The Van Herk Margin Formula (VHMF) and Parker margin formulae were indicated as good candidates for high probabilities of both meeting normal tissue goals and high optimal scores which generally translated to just over 1 mm in GTV to PTV margin.

Conclusion

For single fraction treatment, GTV size is highly significant in predicting failure to meet normal tissue goals whereas whether setup margin was used was not a significant predictor. Setup margin can rationally be applied when fraction number is dictated by clinically indicated metrics regarding GTV size of greater or less than 4 cc. 1 mm is a reasonable practical application of margin added to GTV to ensure physical prescription dose target coverage for most cases when clinically desired based on disease type and intended outcome.

## Introduction

Margins in radiation therapy

Essentially, a setup margin prevents geometric miss of the intended target in part or whole by enlarging the targeted region so that the desired target will fall into the treated area despite instantaneous inaccuracy due to the uncertainty of the system [1,2]. The trade-off is that the new target volume includes more tissue than just the target leading to prescription dose covering some of the surrounding normal tissue which would not have necessarily occurred without the setup margin. For this reason, instead of adding together all the measured uncertainty of the system to create a setup margin that gives 100% confidence in treating the target, the standard deviations of these measured uncertainties are used mathematically to derive setup margin values. This results in high confidence of successful treatment while not expanding the setup margin for all patients to an extent only necessary for very few, worst-case scenario patients who experience the highest possible uncertainty during treatment [2].

Margins in Gamma Knife radiosurgery

Historically, traditional frame-based radiosurgery has not utilized the concept of a setup margin despite identified uncertainty in the system [3]. Largely, this lack of setup margin use has been due to the belief that the uncertainties in the frame-based technique are small enough to be clinically insignificant or less than 1 mm where incorporation of a setup margin would not lead to better local control, but only worse toxicity since larger amounts of normal tissue are now being treated. Additionally, the concerns may be different based on the clinical scenario as the uncertainty related to tumor control probability for metastases or complete obliteration probability for arteriovenous malformations may not apply to all types of targets such as other benign diseases [4]. Now, that significant portions of the treatment system are different from the frameless technique (i.e. immobilization, localization, and monitoring), that argument may not be sufficient nor reasonable as the quantitative value of the new system may be larger and perhaps greater than this perceived/simulated threshold of 1 mm. For instance, the frame immobilization system has been shown to be stable to less than 0.5 mm, but thermoplastic masks have not demonstrated the same effectiveness [5-8]. The conjunction of cone-beam computed tomography (CBCT) localization and motion monitoring may mitigate some of this uncertainty in mask stability, but that has yet to be assessed fully [8,9]. Optimal/rational use of setup margin for frameless Gamma Knife radiosurgery must be investigated with the end goal of providing a final recommendation for quantification and implementation.

In consideration of which margin recipes to apply to the estimation of Gamma Knife radiosurgery margins, several factors should be considered: target size, fraction number, beam penumbra, and isodose prescription line. Those applied to conventional therapy will usually differ in these areas from Gamma Knife radiosurgery. In the performance of Gamma Knife radiosurgery, typically target sizes are small and fraction numbers are low (1-5). The beam penumbra not only is smaller than other treatment modalities but is also anisotropic in nature, meaning the dose fall-off is much steeper in the Z direction than in the X or Y direction leading to a higher cost for uncertainty in this direction. Additionally, the prescription is usually to the 50% isodose line which leads to less of the penumbra being in the vicinity of the edge of the target volume, though choosing this isodose line may be a boon to random uncertainty related to fractionated treatments [10]. Also, as mentioned previously, the additional normal tissue included in setup margins may be of more consequence than that in conventional therapy and should be considered.

The Van Herk Margin Formula (VHMF) is perhaps the most widely known margin formula in radiation oncology, but in its original form, it may not be ideal for the purposes of this work as the recipe was developed for prostate patients with external beam radiation therapy on a linear accelerator and utilizes several assumptions such as biological dose equivalence, infinite fraction number, spherical target, symmetric beam penumbra, and perfect alignment of dose to the target [11]. Other recipes also may be difficult to accurately translate to cranial radiosurgery/therapy as well due to assumptions and differences in the targeted population [12-14]. Consequently, four published recipes have been selected for use in this study: International Council on Radiation Units and Measurements (ICRU) methodology, VHMF with effective uncertainty values, Zhang et al. and Parker et al. [13,15-17]. The ICRU methodology is a generic base minimum recommendation for applying setup margin and therefore shall be applied as a baseline recipe [15]. The VHMF has been shown accurately for one fraction and above when uncertainty values are calculated based on the effective values for the convolution method [13]. The recipe of Zhang et al. is published in both 3D and 1D forms and while it is for a linear accelerator, has been expanded to be more generic. Originally intended for single fraction regimens, however, it would present a maximum possible margin for comparison in multi-fraction cases as well [16,18]. Parker et al. developed their recipe for hypofractionation situations with the assumption of a 20%/mm beam penumbra which is decent for Gamma Knife though perhaps limited still since the penumbra of Gamma Knife are not symmetric and therefore some uncertainties exist in its Gamma Knife application [17].

## Materials and methods

Uncertainty measurement

The total workflow uncertainty was obtained by measurement of the clinical uncertainty of each workflow step for patients treated with frameless immobilization via the Gamma Knife Icon in one to five treatment fractions. Frameless treatment involves immobilization with a thermoplastic mask, localization via a cone-beam CT, and motion management via an infrared camera and fiducial marker. The details of this technique and the uncertainty measurement can be reviewed in the work accepted for publication in the Journal of Applied Clinical Medical Physics [19].

Margin application

This study sought to experimentally apply setup margins to frameless Gamma Knife treatment planning in order to observe quantitatively the effects on the dose distribution and discern a rational approach to setup margin application for frameless radiosurgery with the Gamma Knife Icon. Select published margin recipes (Table 1) were used to provide setup margins recommendations that were then applied to targets of 30 previously treated patients [11,15,17-18,20].

**Table 1:** Margin recipes to be used in this study *Using effective values of Σ, σ [13,20] **Simplified formula [16,18] Prescription radiation dose is abbreviated as "Rx" above

Recipe	Authors	Types of Uncertainty	Fraction Number	Target Type	CTV Coverage	Formula
ICRU	ICRU [1]	Systematic and Random	Not Defined	Not Defined	Not Defined	√(Sys^2^+Rand^2^)
VHMF Mod*	Van Herk et al. [11]	Systematic and Random	≥ 1	Prostate	95% of Rx for 90% of patients	2.5 Σ_eff_ + 1.64 (σ_eff_-σ_p_)
Zhang	Zhang et al. [16]	Systematic and Residual	1	Brain Metastasis	100% of Rx for 95% of patients	2.787β**
Parker	Parker et al. [17]	Systematic and Random	5 to 30	Cranial Targets	95% of Rx for 99% of patients	Systematic + 1Σ + 1σ

These recipes were compared based on the change in relative target volume after application and the relative increased probability of actually intended target dose coverage. Margin size was calculated based on uncertainty measurements made during an in-house study on uncertainty during frameless Gamma Knife treatment. Once margins were applied in the MIM Radiation Oncology software, treatment plans were developed on the new planning target volumes (PTVs) to attempt to meet similar planning goals as the original clinical plans such as coverage, conformity, selectivity, and gradient index, which are defined in the literature [3].

Margin recipe scoring

After the test treatment plans were developed, they were evaluated on whether clinically acceptable normal tissue goals were met. Table 2 shows the primary normal tissue tolerances used in this study and their respective references. ​​​

**Table 2:** Normal tissue constraints utilized in the evaluation of all treatment plans ^a^The brain volume constraint listed is per lesion, the volume increases to 30 cc for multi-lesion plans ^b^The volume dose is marginal for multi-fraction but includes gross tumor volume (GTV) for single fraction regimens Refs. [21-24]

Normal Tissue Constraints (Doses in Gy)								
	Single Fraction		3 Fractions		4 Fractions		5 Fractions	
Structure	Volume	Max	Volume	Max	Volume	Max	Volume	Max
Brain^a^	V12<10cc	None	V16.5<20cc^b^	None	V18.4<20cc^b^	None	V20<20cc^b^	None
Brainstem	V10<0.5cc	15	V18<0.5cc	23.1	V20.5<0.5cc	27	V23<0.5cc	31
Cord	V10<0.35cc	15	V18<0.35cc	23.1	V22<0.35cc	26.5	V26<0.35cc	30
Optics	V8<0.2cc	12	V15.3<0.2cc	20	V19<0.2cc	22.5	V23<0.2cc	25
Cochleae	None	9	None	17.1	None	21	None	25

The three and four fraction tolerance for brain tissue was derived from the five-fraction tolerance using the linear-quadratic formula and is assessed in the same way as the five-fraction tolerance. It is important to note that for 5 fraction plans, the tolerance comes from phase two data in which the “normal” (non-gross tumor volume [non-GTV]) brain volume was assessed and therefore the GTV is not included in the 20 cc tolerance [21,22]. Each target was evaluated independently but still based on the cumulative dose distribution, meaning that the volume of the normal brain at the critical dose was assessed for each lesion to meet Table 3 tolerance and the cumulative for all lesions was allowed to be higher up to 30 cc. This is in keeping with evidence and clinical trials at the time of this study that the largest lesion has been found to be statistically significant as a predictor of post-radiation toxicity whereas the number of lesions has not been [23]. 120 new treatment plans for 30 patients (one plan per setup margin per patient) were generated meeting similar coverage and conformity indices compared to the original plan with no margin. All five treatment plans (including original without margin) for each patient were evaluated based on doses to critical structures and volume of critical brain dose-treated. If any critical structure was over Table 2 max dose tolerances or volume of the brain above the critical dose, then that plan failed. Then, each plan received an optimal score based on the amount of coverage ensured and the extent to which normal tissue tolerances were met as discussed below.

**Table 3:** Summary of how treatment plans were scored after the use of each margin formula *Target Coverage Score negated to zero based on any normal tissue dose failure

Optimal Margin Scoring System		
Category	Plan Value	Scoring
Target Coverage	0, 1, 2, 3, 4	+ Value x 10
Brain Dose Volume	Per cc over 10 cc (20 cc for Multi-Fx lesions)	- 10
	> 10cc (20 cc for Multi-Fraction lesions)	- Target Coverage Score*
	< 10 cc (20 cc for Multi-Fraction lesions)	0
	< 8.4 cc (16.8 cc for Multi-Fraction lesions)	+ 10
	< 7.9 cc (15.8 cc for Multi-Fraction lesions)	+ 20
Brainstem, Cord, Chiasm, and Optic Nerves	Max Dose > table 6 max dose	- Target Coverage Score*
	Max dose < table 6 max dose	0
	Max dose < table 6 volume max dose	+ 10
Cochleae	Max Dose > table 6 max dose	- Target Coverage Score*
	Max dose < table 6 max dose	0

Optimal score

It can be seen in Table 1 that each setup margin recipe was developed with different mathematical goals and therefore the margins can be ranked in order of coverage confidence provided. This is also perhaps intuitive upon review of the amount of setup margin that is indicated through each margin calculation. For all cases, the Zhang recipe provides the highest level of confidence that the target will be covered despite uncertainty, while the ICRU provides the lowest out of the four margin recipes. The VHMF and Parker formulas always fall into the middle for patients, but their ranking respective to each other is dependent on fraction number. Though the Parker recipe nominally provides higher coverage confidence, it was developed with five fraction regimens in mind and therefore its nominal coverage confidence no longer applies when the fraction number decreases below this number [17]. When using the effective values of uncertainty during margin calculation for the VHMF as we are in this study, the VHMF compensates somewhat for the number of fractions in the treatment and should still provide close to its nominal coverage confidence goal [13]. Taking this into account, for treatments involving only one to four fractions, the VHMF ranks higher, but the Parker becomes higher ranking compared to VHMF for five fraction treatments. For the base calculation of the optimal score, the rank position for target coverage was multiplied by 10 (i.e. Zhang gets 4 which becomes 40).

In addition to coverage concerns, potential added toxicity also needed to be considered in the optimal score. Clinically identified normal tissue tolerances shown to lead to lower toxicity risk in clinical studies were utilized to increase a margin’s optimal score for a given patient. For example, though for a single fraction treatment, V12 of the normal brain has normally been constricted to 10 cc for an individual lesion, others have shown that reducing this volume even further decreases toxicity risk. For example, the traditional constraint may indicate a 25%-30% risk of necrosis (asymptomatic and symptomatic) while 8.4 cc and 7.9 cc may indicate a 15% and 10% risk, respectively [23]. For non-brain tissue tolerances, the volumetric constraint was modified to a maximum dose constraint and when met added to the optimal score for a given margin and patient. The scoring criteria for the optimal score are shown in Table 3.

The scoring system is somewhat arbitrary, but it is based on published literature for dose constraints and the coverage ensured by the various margin recipes [11,16,17,21-24]. Alternatively, tumor control probability or normal tissue complication probability models could have been included, but though developed, these models have endured scrutiny and even controversy regarding clinical validation and as a result, have not yet been widely accepted [25]. This scoring system utilizes known and trusted normal tissue tolerances and discriminates between treatment plans based on the ability to meet those tolerances while ensuring target coverage. Plans without margins only receive credit for how well normal tissue criteria are met and plans incorporating margins only receive credit for increased coverage when normal tissue constraints are still met. The system is simple, but effective to differentiate between treatment plans incorporating different margin recipes leading to different dose distributions.

Statistical analysis

All statistics were performed with SPSS 24 Statistical Analysis software (IBM Corp., Armonk, NY, USA). New treatment plans were developed for each margin on all targets and each plan received both pass or fail and an optimal score. Bivariate analysis determined factors correlated with passing and high scores and then logistic regression was used to model those factors to predict a pass/fail.

## Results

Margin calculation

The uncertainty values in the X, Y, and Z directions were utilized to calculate setup margins via the aforementioned ICRU, VHMF, Parker, and Zhang methodologies. The resulting values can be seen in Table 4.

**Table 4:** Calculation of direction-dependent setup margins and relevant values Fraction is abbreviated as “Fx” in some instances within the table

		X (mm)	Y (mm)	Z (mm)
Systematic Error Mean		0.25	0.34	0.48
Systematic Error Standard Deviation (SD)		0.43	0.47	0.44
Effective Systematic SD	1 Fx	0.54	0.57	0.64
	3 Fx	0.47	0.50	0.52
	4 Fx	0.46	0.49	0.50
	5 Fx	0.45	0.49	0.49
Random/Residual Error Mean		0.02	0.06	0.26
Random/Residual Error SD		0.33	0.32	0.45
Effective Random/Residual SD	1 Fx	0.00	0.00	0.00
	3 Fx	0.27	0.26	0.37
	4 Fx	0.29	0.28	0.39
	5 Fx	0.30	0.29	0.41
Penumbra (45-25%)*		1.13	1.13	0.53
ICRU		0.54	0.57	0.64
VHMF 1 Fraction		1.35	1.42	1.59
VHMF 3 Fractions		1.17	1.25	1.29
VHMF 4 Fractions		1.14	1.23	1.25
VHMF 5 Fractions		1.13	1.22	1.22
Parker		1.01	1.13	1.37
Zhang 1D		1.40	1.52	1.79

Note that the VHMF was the only formula that varied with a number of fractions based on the usage of effective values of Σ, σ which vary with fraction number. It should also be noted that despite the fast fall-off of the Gamma Knife dose distribution, the random error was so small that it was encompassed by the penumbra in all directions. An appendix on individual setup margin calculation per recipe can be provided upon request.

Statistical analysis on margin application (pass/fail)

The frequencies for pass/fail and descriptive statistics for the optimal score can be seen in Table 5.

**Table 5:** Summary of treatment plan analysis for each margin recipe and original plans (n = 150 plans)

		Normal Tissue Dose Exceeded			Optimal Score			
		No	Yes	Total Plans	Mean	Min	Max	St. Dev.
Setup Margin Recipe	None	27	3	30	52.3	0	60	15.2
	ICRU	25	5	30	58.3	0	70	19
	VHMF	22	8	30	61	0	90	32.2
	Parker	22	8	30	62.7	0	90	29.5
	Zhang	19	11	30	65	0	100	39.8

A Pearson Chi-Square test indicated a non-significant relationship between plans utilizing any margin recipe and the original plans for prediction of pass/fail with Pearson Chi-Square (4) = 7.081, p = 0.132; therefore, the use of any margin was not statistically predictive of plan failure. Using the point biserial correlation, the number of fractions was the only moderate correlation identified as having a statistically significant relationship with a pass/fail score of r = -0.398, p < 0.001, indicating that as the number of fractions goes up the likelihood of plan failure potentially goes down (Table 6). 

**Table 6:** All moderate to strong relationships identified to be statistically significant in correlation analysis after margin application in various scenarios (no significant relationships found in correlation for multi-fraction, pass/fail)

Scenario	Variable	Coefficient	p-value	Strength	Significant			95% CI	N
All Plans, Pass/Fail	Number of Fractions	-0.398	< 0.001	Moderate	Highly			-0.525, -0.254	150
All Plans, Optimal Score	Patient Head Lateral Diameter	-0.321	< 0.001	Moderate	Highly			-0.458, -0.169	150
	Number of Fractions	0.362	< 0.001	Moderate	Highly			0.214, 0.494	150
Single Fraction, Pass/Fail	Patient Weight	0.338	0.002	Moderate	Yes			0.128, 0.519	80
	Patient Head Lateral Diameter	0.4	< 0.001	Moderate	Highly			0.198, 0.570	80
	Number of Targets	-0.352	0.001	Moderate	Yes			-0.531, -0.143	80
	Number of Central Targets	-0.411	< 0.001	Moderate	Highly			-0.578, -0.210	80
	Largest GTV Volume	0.541	< 0.001	Strong	Highly			0.365, 0.680	80
	Total GTV Volume	0.504	< 0.001	Strong	Highly			0.320, 0.652	80
	Total PTV Volume	0.528	< 0.001	Strong	Highly			0.349, 0.670	80
Single Fraction, Optimal Score	Patient Weight	-0.369	0.001	Moderate	Yes			-0.545, -0.162	80
	Patient Head Lateral Diameter	-0.463	< 0.001	Moderate	Highly			-0.620, -0.271	80
	Number of Targets	0.376	0.001	Moderate	Yes			0.170, 0.550	80
	Number of Peripheral Targets	0.300	0.007	Moderate	Yes			0.086, 0.488	80
	Number of Central Targets	0.402	< 0.001	Moderate	Highly			0.200, 0.571	80
	Largest GTV Volume	-0.662	< 0.001	Strong	Highly			-0.770, -0.518	80
	Total GTV Volume	-0.644	< 0.001	Strong	Highly			-0.757, -0.494	80
	Total PTV Volume	-0.613	< 0.001	Strong	Highly			-0.734, -0.454	80
Multi-Fraction, Optimal Score	Patient Weight	-0.349	0.003	Moderate		Yes	-0.540, -0.124		70
	Patient BMI	-0.373	0.001	Moderate		Yes	-0.559, -0.151		70
	Average Sphericity (Equivalent Sphere/Max Diameter)	0.34	0.004	Moderate		Yes	0.114, 0.532		70

Choosing a multiple fraction regimen appears to greatly reduce the chance of failing to meet normal tissue criteria whether using margin or not. Due to these results, the data were grouped based on the single fraction or multiple-fraction and re-analyzed within these groups with the same correlational tests. 

Single Fraction Only (Pass/Fail)

**Figure 1:**
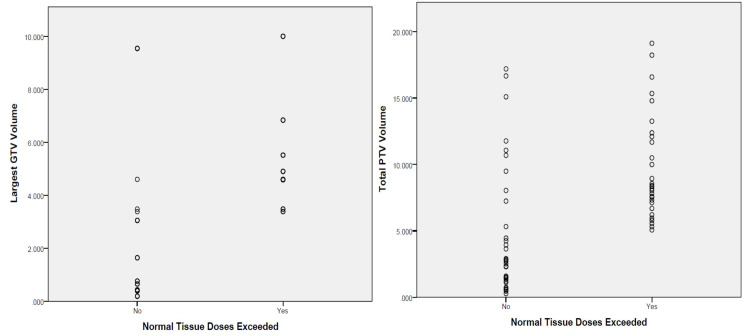
Plots of largest GTV volume and total PTV volume versus Pass/Fail (Normal Tissue Dose Exceeded). A distinction can be seen at 4 cc and 5 cc for the GTV and PTV, respectively.

A Pearson Chi-Square test indicated a non-significant relationship between plans utilizing any margin recipe and the original plans for prediction of pass/fail with Pearson Chi-Square (4) = 5.521, p = 0.238; therefore, the use of any margin was not statistically predictive of plan failure as even the original plans failed to meet normal tissue doses in three out of 16 cases for the single fraction cases. Statistically significant relationships were discerned with the point biserial correlation. Lateral head diameter (r = 0.4, p < 0.001), number of central targets (r = -0.411, p < 0.001), and target number (r = -0.352, p = 0.001) were indicated as moderate correlations. Strong correlations were found with largest GTV (r = 0.541, p < 0.001), total GTV (r = 0.504, p < 0.001), and total PTV (r = 0.528, p < 0.001) (Table 6). Upon further review of the data for single fraction plans, it seemed that failures became much more common once the GTV or PTV reached a certain size just under 4 cc and 5 cc, respectively (Figure 1).

Multi-Fraction Only (Pass/Fail)

A Pearson Chi-Square test indicated a significant relationship between margin recipe and the pass/fail score with Pearson Chi-Square (4) = 12.537, p = 0.014. Only the Zhang margin recipe led to failure to meet normal tissue goals in three out of 14 cases, while the other recipes passed for all plans (as did the original plans with no margin). No other factors were found to be significant with the pass/fail score for multi-fraction cases, therefore the correlation results are not shown in Table 6.

Statistical Analysis on Margin Application (Optimal Score)

Using the Pearson correlation test, statistically significant, moderate relationships were found with optimal score in lateral head diameter (r = -0.321, p < 0.001) and number of treatment fractions (r = 0.362, p < 0.001), as indicated in Table 6. Considering all the data together, a one-way ANOVA test indicated that there was no statistical significance, F (4,149) = 0.869, p = 0.484, in the choice of margin recipe (ICRU, VHMF, Parker, or Zhang) or none at all and the resulting optimal score for all data together. Based on the strongest correlation being the number of fractions, the data was again dummy coded based on single or multi-fraction and analyzed.

Single Fraction Only

Again, a one-way ANOVA yields a non-significant result for this group of data when considering margin choice including none, F (4, 79) = 0.086, p = 0.987. Data for treatment plans without margin were again excluded, Pearson correlation indicated moderate relationships with weight (r = 0.369, p = 0.001), head diameter LR (r = -0.463, p < 0.001), number of peripheral targets (r = 0.3, p = 0.007), number of central targets (r = 0.402, p < 0.001), and overall target number (r = 0.376, p = 0.001). Similar to the pass/fail score, strong relationships were again identified with largest GTV (r = -0.662, p < 0.001), total GTV (r = -0.644, p < 0.001), and total PTV (r = -0.613, p < 0.001) (Table 6). Based on this data, it seems that target volume is more of a predictor of the optimal score value for a single fraction plan than whether margin is added or not, and though PTV is dependent on margin, optimal score may perhaps be more dependent on the original GTV .

Multi-Fraction Only

For multi-fraction plans, a one-way ANOVA was used for the relationship: None, M = 57.14 (6.112), ICRU, M = 65 (7.596), VHMF, M = 75 (11.602), Parker, M = 80.71 (11.411), and Zhang, M = 80 (26.018), yielding a highly statistically significant result of F (4, 69) = 7.051, p < 0.001. Furthermore, based on the LSD post hoc analysis, all but the ICRU recipe shows a statistically significant improvement in optimal score mean over using no margin at all. The Parker recipe is statistically significant from all but the VHMF and Zhang recipe, but the VHMF was not significantly different from the ICRU recipe and the Zhang demonstrates more variation in the optimal score based on the standard deviation. Additionally, some statistically significant correlations were found relating to optimal score such as moderate correlations: weight (r = -0.349, p = 0.003), body mass index (BMI) (r = -0.373, p = 0.001), and average GTV sphericity (r = 0.34, p = 0.004) and are recorded in Table 6.

Summary

It appears that the choice of margin recipe is not a significant predictor of a decrease in the plan quality based on the normal tissue doses and optimal score presented here unless limited to multi-fraction treatment plans. For a single fraction, it appears that the GTV and subsequently, PTV size have a stronger influence on the outcome of plan quality. For multi-fraction plans, the Zhang recipe was identified as a potential predictor of failing to meet normal tissue goals while the ICRU recipe failed to show a statistically significant increase in optimal score from using no margin. A practical outcome of this study may be the GTV and PTV volume thresholds to consider of around 4 cc and 5 cc, respectively.

## Discussion

When all 150 plans were analyzed together and when grouped as only single fraction plans, the margin recipe was not significant in predicting the quality of plans but was significant for multi-fraction plans. The results of this study indicate that even the choice to use a margin is not the strongest predictive factor in whether a plan will be clinically optimal or avoid toxicity for patients. Initially, the strongest correlation was the number of fractions. As the number of fractions goes up, the chances that critical structures will be spared and that optimal scores will be high all go up. This concept is already intuitive and utilized often in the clinic when targets are perilously close to critical structures. However, in the realm of Gamma Knife radiosurgery, the idea of fractionation may also provide room to include setup margins in the treatment planning so that the targets are covered optimally with less risk to critical structures. This is not to say that one has to fractionate to use setup margins as they were applied in this study for single fraction cases also with spared normal tissues and high optimal scores in many cases. However, traditional decisions of when to fractionate may need review. This concept has been validated with clinical data to some extent where it was seen that according to a meta-analysis of 24 different trials, radionecrosis risk decreases with a number of fractions while maintaining local control for lesions greater than 2-3 cm in diameter (4-14 cc) and above [26].

The results of this study seem to corroborate clinical results showing a benefit to fractionation and increasing the optimal score (high probability of coverage plus toxicity avoidance) and avoiding critical structure tolerances [24]. Application of setup margins may be more difficult for single fraction when GTV size is nearing 4 cc (which already indicates a higher probability of normal tissue toxicity), fractionation should be considered to be able to safely add setup margin. For single fraction plans, no significant difference was found between the margin recipes, so it may be up to the clinician’s preference as to whether they would rather err on the side of conservative target coverage or risk missing the target to be sure to spare more brain tissue. For multi-fraction plans, the Zhang recipe was found to cause failures in some cases which is not surprising since it is the largest margin and was developed for single fraction plans. The ICRU recipe failed to differentiate significantly from the use of no margin, so perhaps one's choice of either the Parker or the VHMF might be acceptable, though, with the use of effective uncertainty values, VHMF is designed to handle changes in fractionation better. Truly, the amount of setup margin for either is very similar and they are not statistically different from each other. Also, a more practical approach might be to choose a value close to the recommended amount from this work, say 1 mm, for instance, to implement as setup margin, but which is similar to that predicted by the VHMF or Parker formulae (Table 4). Utilizing a similar approach of 1 mm for PTV margin (1.5 mm superior and inferior), a recent phantom study demonstrated acceptable dose delivery even under the worst-case scenario for frameless radiosurgery with Gamma Knife Icon [27]. In any situation, rational margin application should seek to balance the risk of normal tissue injury from radiation treatment with the risk of injury to the same tissue due to uncontrolled disease [28].

Opportunities for further study

In addition to margin recipe and target sizes, other relationships were identified as potentially related to plans passing or failing and optimal scores. These factors warrant further study and review to elucidate reasons that these factors might be predictors of higher quality treatment plans as well truly whether those results are repeatable. Perhaps one of the most interesting factors is the left-right head diameter in that it was highly significant in several cases as a potential predictor of weak to moderate strength. Perhaps this is due to the physics of dose distribution for these cases where a lower percent depth dose for each of the 192 beams means that the dose is more spread out for these cases leading to higher volumes of critical brain dose.

## Conclusions

Historically, the application of setup margins has not been standard with Gamma Knife radiosurgery, but now that immobilization, localization, and motion management/monitoring methods are different with frameless radiosurgery, the level of uncertainty is different. The significance of this study lies in exploration of optimal/rational implementation of setup margin recipes considering not only geometric accuracy, but also avoidance of unnecessary normal tissue toxicity. The results of this study have important implications on optimal clinical treatment protocol with Gamma Knife radiosurgery and considerations have been stated as to how the findings of this study might be used if desired. It should be noted that any application of setup margin should be rational in that the risk of continued normal tissue injury due to tumor underdosing must be balanced with the risk of normal tissue injury due radiation dose. The results of this work indicate methodology to recommend patient specific use of certain margin recipes as well as rational consideration of setup margin during frameless Gamma Knife treatment.
